# Supplementary figures and images for: Genome-Wide Analysis of the YABBY Transcription Factor Family in Rapeseed (Brassica napus L.)
Source: Genes (Basel). 2021 Jun 27;12(7):981. doi: 10.3390/genes12070981 (PMC8306101; doi:10.3390/genes12070981)

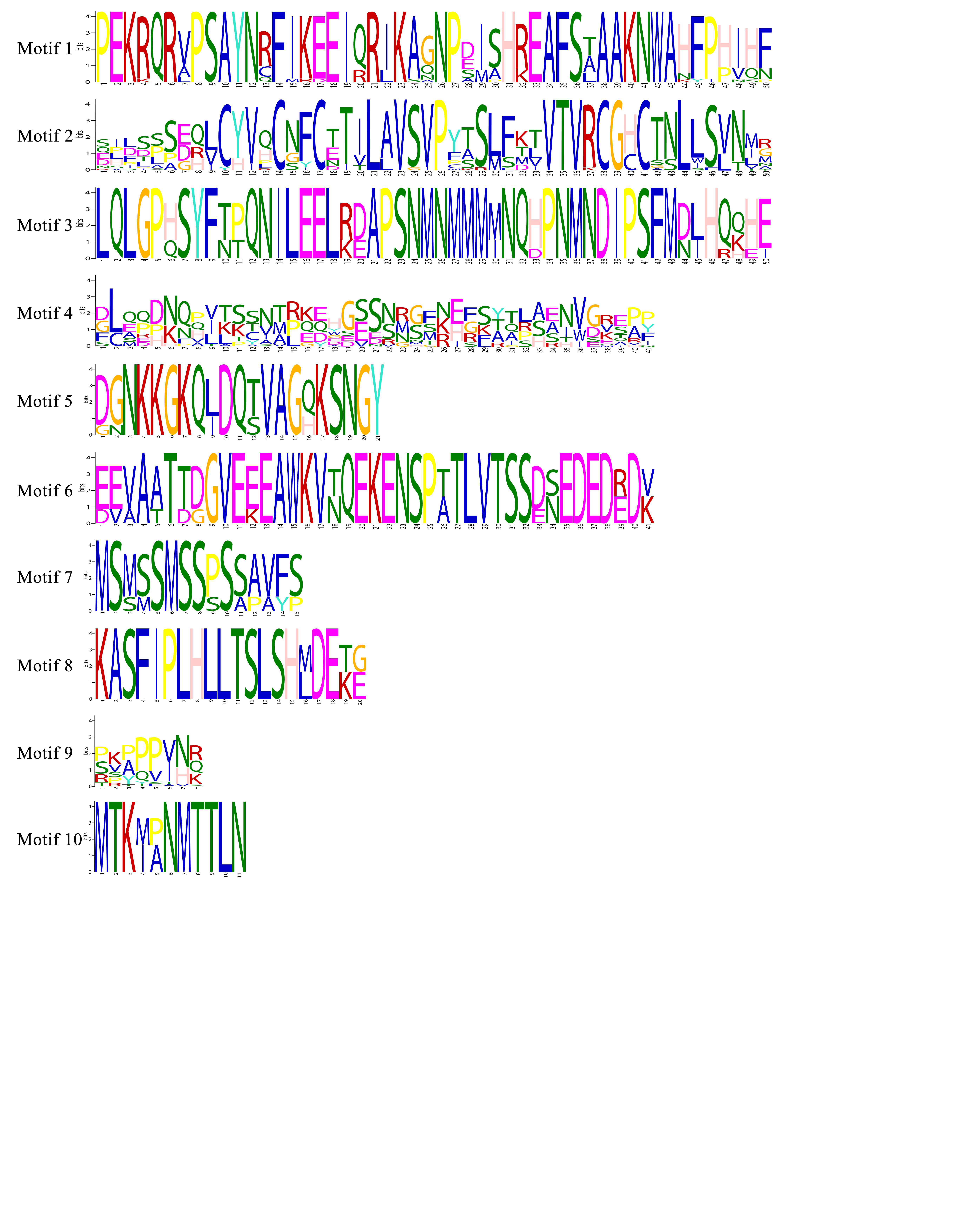

Supplement: Supplementary file 1 [file genes-12-00981-s001.zip › ╕╜╝■/Figure S1.png]
